# Poor glycemic control and smoking and drinking history rather than bacterial virulence contribute to the development of invasive *Klebsiella pneumoniae* liver abscess: a case–control study in Northeast China

**DOI:** 10.3389/fmicb.2025.1650703

**Published:** 2025-08-26

**Authors:** Jingjing Chen, Xinyi Wang, Qihui Wang, Sufei Tian, Fushun Li, Ruixuan Wang, Zhihui Chang, Yunzhuo Chu

**Affiliations:** ^1^Department of Laboratory Medicine, National Clinical Research Center for Laboratory Medicine, The First Hospital of China Medical University, Shenyang, China; ^2^Research Unit of Medical Laboratory, Chinese Academy of Medical Sciences, Beijing, China; ^3^Department of Radiology, Shengjing Hospital of China Medical University, Shenyang, China

**Keywords:** liver abscess, *Klebsiella pneumoniae*, invasive, virulence, risk factor, fasting blood glucose

## Abstract

**Objective:**

This study aimed to investigate the clinical and microbiological characteristics of invasive and noninvasive *Klebsiella pneumoniae* liver abscesses (KPLAs) and elucidate the risk factors for invasive KPLA.

**Methods:**

We conducted a case–control study involving 50 patients with invasive KPLA and 50 patients with noninvasive KPLA from two medical centers between 2019 and 2024. Demographic and clinical data were collected for all patients from the hospital medical records system. Univariate and multivariate analyses were then performed to compare the characteristics of invasive and noninvasive KPLAs. In addition, antimicrobial resistance testing and whole-genome sequencing were performed for 50 *K. pneumoniae* strains from one medical center.

**Results:**

The comparison of patients with invasive and noninvasive KPLAs revealed that diabetes mellitus, smoking history, drinking history, smaller maximum diameter of abscess, neutrophil count, fasting blood glucose, blood urea nitrogen, and length of hospital stay were independent risk factors for invasive KPLA. Phylogenetic analysis revealed that *K. pneumoniae* strains from patients with invasive and noninvasive KPLAs were intermingled. ST23 with K1 serotype was the predominant sequence type (66.0%), followed by ST65 with K2 serotype. Multilocus sequence types, capsular serotypes, antimicrobial resistance patterns, virulence genes, and SNPs of *K. pneumoniae* strains isolated from patients with invasive and noninvasive KPLAs showed no significant differences between the two patient groups.

**Conclusion:**

Overall, our results indicate that patients’ contaminant conditions, such as poor glycemic control, smoking history, and drinking history, rather than bacterial virulence, contribute to the development of invasive KPLA.

## 1 Introduction

Pyogenic liver abscess (PLA) is a life-threatening infectious disease caused by the invasion of bacteria into the liver, resulting in the formation of single or multiple purulent infection foci. *Klebsiella pneumoniae* liver abscess (KPLA) was first reported in 1986 in Taiwan, China (Liu et al., 1986). Recently, it has been increasingly reported in Asia and has been designated an emerging infectious disease globally during the past two decades (Siu et al., 2012). The most common clinical manifestations in patients with KPLA are fever, chills, and abdominal pain, with a mortality rate of approximately 3.8% (Lee et al., 2020). KPLA can also cause extrahepatic migratory infections, such as endophthalmitis, lung abscess, renal abscess, and even meningitis, which have been reported in approximately 10–16% of cases (Wang et al., 1998), with lungs and eyes being the common sites of invasive infections (Sun et al., 2021). KPLA complicated with extrahepatic metastatic infection is defined as invasive *K. pneumoniae* liver abscess syndrome (IKPLAS). Invasive KPLA is associated with increased rates of mortality and disability as well as higher lengths of hospital stay and cost of hospitalization compared with noninvasive KPLA, posing a serious threat to human health. For example, KPLA with endophthalmitis is often accompanied by decreased vision or eye pain, and it can also progress to permanent vision loss (Hussain et al., 2020).

Hypervirulent *K. pneumoniae* (hvKP), the major pathogen responsible for KPLA, differs significantly from classical *K. pneumoniae* (cKP) in terms of its biological characteristics and the clinical features of the diseases it causes. HvKP strains exhibit a hypermucoviscous phenotype and K1 or K2 serotype; moreover, they harbor multiple hypervirulence determinants related to capsular polysaccharides, lipopolysaccharides, and siderophores (e.g., *peg-344*, *iroB*, *iucA*, *rmpA*, and *rmpA2*) (Zhu et al., 2021). hvKP strains are usually susceptible to commonly used antibiotics (Kocsis, 2023); however, extended-spectrum β-lactamase-producing and carbapenem-resistant hvKP are increasingly reported since 2015 (Morales-Leon et al., 2021; Lei et al., 2024). The predominant sequence type that causes KPLA worldwide is ST23 (Sohrabi et al., 2022). Unlike cKP, which is a major nosocomial pathogen, hvKP usually causes infections in the community in healthy people or individuals with diabetes mellitus. HvKP has been associated with various clinical manifestations, such as liver and renal abscesses and bacteremia (Chen et al., 2023).

A few studies have investigated clinical characteristics of IKPLAS, which reported different risk factors for invasive KPLA, such as sequential organ failure assessment (SOFA) score ≥ 4, breathlessness, abscess size, diabetes mellitus, thrombocytopenia, etc. (Chen et al., 2006; Yoon et al., 2014; Chang et al., 2015; Mukherjee et al., 2022; Gu et al., 2025). Additionally, results obtained from different studies were also inconsistent. For example, smaller abscess size was reported to be an independent risk factor for invasive KPLA (Shin et al., 2013; Chang et al., 2015), whereas another study indicated that patients with larger abscess size was at a greater risk of metastatic KPLA (Mukherjee et al., 2022). This may be due to small sample size of patients with invasive KPLA included in the studies. Besides, the role of bacterial virulence in the development of IKPLAS remains poorly characterized. In the present multicenter study, we investigated the clinical characteristics of patients with invasive and noninvasive KPLAs. In addition, we compared the microbiological and genomic characteristics of *K. pneumoniae* isolates isolated from patients with invasive and noninvasive KPLAs. Our results may aid in the early identification of patients with KPLA at increased risk of invasiveness, thereby improving clinical management and enabling personalized treatment.

## 2 Materials and methods

### 2.1 Study participants

This retrospective case–control study was performed at two medical centers in Northeast China: The First Hospital of China Medical University and Shengjing Hospital of China Medical University. The inclusion criteria of KPLA were: (1) imaging showing one or more lesions in the liver; (2) Positive culture for *K. pneumoniae* from pus, fluid, or blood culture samples. The exclusion criteria was liver abscess secondary to liver surgery, liver tumors, and other liver diseases. Invasive KPLA was defined as KPLA accompanied with extrahepatic metastatic infections at other body sites indicated by imaging during the same admission. Whereas noninvasive KPLA was defined as KPLA without bloodstream infection or extrahepatic infections. The clinical groups were determined by two clinicians independently. In cases of disagreement, a third clinician was consulted.

Fifty patients were enrolled in the invasive KPLA group from January 2019 to December 2024. The specimen types with positive *K. pneumoniae* cultures were: blood (17), pus (13), fluid (9), both blood and pus (8), both blood and fluid (3). Another 50 patients with KLPA were randomly selected to form the noninvasive KPLA group. Among them, 26 and 24 patients had positive *K. pneumoniae* cultures in pus and fluid specimens, respectively. Patients with invasive KPLA had the following extrahepatic infections: lung abscess (22), endophthalmitis (16), endophthalmitis and lung abscess (5), lung abscess and renal abscess (3), renal abscess (2), endophthalmitis and osteomyelitis (1), endophthalmitis and brain abscess (1). Fifty patients were enrolled in each medical center respectively, including 25 patients with invasive KPLA and 25 patients with noninvasive KPLA.

### 2.2 Microbiological methods

Fifty *K. pneumoniae* strains from 25 patients with invasive KLPA and 25 patients with noninvasive KPLA recovered from one medical center (The First Hospital of China Medical University) were subjected to microbiological testing and whole-genome sequencing. Suspicious *K. pneumoniae* strains growing on Columbia blood agar or MacConkey agar were identified by Vitek 2 or Matrix-Assisted Laser Desorption/ Ionization Time of Flight Mass Spectrometry (MALDI-TOF, bioMérieux, France). Additionally, antimicrobial resistance profiles of the strains were determined using Vitek 2 and interpreted according to the criteria of Clinical and Laboratory Standards Institute (CLSI) M100 (35th Edition). *E. coli* ATCC 25922 and *P. aeruginosa* ATCC 27853 were used for quality control of antimicrobial susceptibility testing. We also performed the string test, a well-known test to detect the mucoviscous phenotype of *K. pneumoniae*, with a viscous string of >5 mm as positive (Chen et al., 2021). *K. pneumoniae* NTUH-K2044 and ATCC 700603 were used as positive and negative controls, respectively.

### 2.3 Clinical data collection

We collected the following demographic and clinical data from the hospital medical records system using a standardized form: demographics (age and sex), history of cigarette smoking and alcohol drinking, underlying conditions (hypertension, diabetes mellitus, etc.), clinical symptoms (fever, chill, vomiting, and abdominal pain), laboratory test results (white blood cell count [WBC], neutrophil count, lymphocyte count, hemoglobin, platelet count [PLT], aspartate transaminase [AST], alanine aminotransferase [ALT], albumin, total bilirubin, blood glucose, HbA1c, blood urea nitrogen [BUN], creatinine [Cr], prothrombin time [PT], procalcitonin [PCT], C-reactive protein [CRP], etc.), and clinical outcomes (length of hospital stay, admission to intensive care unit [ICU], septic shock, and in-hospital deaths), shown in Supplementary Table S1.

### 2.4 Whole-genome sequencing and data analysis

Whole-genome sequencing was performed in Personalbio company (Shanghai, China). DNA was extracted from bacteria that were grown overnight with the CTAB extraction assay previously described (Wilson, 2001). Then TruSeq^™^ DNA Sample Prep Kit was used to construct the genomic library following the manufacturer’s operating procedures. Then resequencing was performed using an Illumina NovaSeq platform, with a sequencing mode of paired-end, 2 × 150 bp. Sequencing data were concatenated using SPAdes v3.15.4.^1^ In addition, pathogenwatch^2^ was used to determine the virulence gene score, resistance gene score, ST type, wzi type, and K/O type of bacterial strains. GATK v3.8 was used to detect single nucleotide polymorphisms (SNP), and a core-genome SNP-based phylogenetic tree was then constructed using the Maximum Likelihood algorithm implemented in the FastTree v2.1.11. The reliability of the phylogenetic tree was evaluated using bootstrap analysis with 1,000 replications. Finally, VFDB^3^ was used to analyze the virulence genes of the *K. pneumoniae* strains.

### 2.5 Statistical analysis

SPSS 20.0 was used for statistical analysis. Quantitative data were expressed as mean ± standard deviation or median (interquartile range) and compared using Student’s t-test or nonparametric test. In contrast, qualitative data were represented as rates and compared using chi-square test or Fisher’s test. After univariate analysis, variables with *p* < 0.1 rather than *p* < 0.05 were included in the multivariate analysis, as *p* < 0.1 reduced the risk of missing important variables (Sauerbrei et al., 2007). Multivariate binary logistic regression analysis was used to determine the risk factors associated with invasive KPLA after multiple imputations of the missing data. Statistical significance was determined at *p* < 0.05.

## 3 Results

### 3.1 Univariate analysis of the clinical characteristics of patients with invasive and noninvasive KPLAs

Univariate analysis revealed a high percentage of smoking history and diabetes mellitus among patients with invasive KPLA (Table 1). 68.0% of patients with invasive KPLA had a history of diabetes mellitus or were recently diagnosed with diabetes mellitus. Moreover, patients with invasive KPLA had higher neutrophil counts, fasting blood glucose levels, and BUN levels than those with noninvasive KPLA. Patients with invasive KPLA tended to have longer hospital stay than those with noninvasive KPLA.

**Table 1:** Comparison of the clinical characteristics of patients with invasive and noninvasive KPLAs.

Characteristic	Invasive KPLA (*n* = 50)	Noninvasive KPLA (*n* = 50)	*p* value
Age^&^	56.2 (14.1)	59.5 (14.0)	0.23
Sex			
Male	36/50 (72.0)	28/50 (56.0)	0.096
Underlying or concomitant conditions			
Smoking history	20/49 (40.8)	6/46 (13.0)	0.002*
Drinking history	18/48 (37.5)	9/46 (19.6)	0.055
Diabetes mellitus	34/50 (68.0)	17/50 (34.0)	0.001*
Hypertension	16/50 (32.0)	11/50 (22.0)	0.26
Highest temperature (°C)^&^	39.3 (0.9)	39.0 (0.9)	0.14
Clinical symptoms			
Fever	46/50 (92.0)	49/50 (98.0)	0.36
Chill	31/50 (62.0)	29/50 (58.0)	0.68
Abdominal pain	6/50 (12.0)	12/50 (24.0)	0.12
Vomit	10/50 (20.0)	13/50 (26.0)	0.48
Multiplicity of the abscess			
Single	29/46 (63.0)	32/50 (64.0)	0.92
Multiple (≥2 abscesses)	17/46 (37.0)	18/50 (36.0)	0.92
Site of the abscess			
Right hepatic lobe	32/48 (66.7)	29/49 (59.2)	0.45
Left hepatic lobe	9/48 (18.7)	10/48 (17.0)	0.84
Both	6/48 (12.5)	9/48 (18.4)	0.42
Maximum diameter of the abscess, cm^&^	5.8 (2.5)	6.7 (2.4)	0.088
Laboratory examination			
Neutrophil, ×10^9^/L^&^	11.7 (6.3)	9.2 (4.0)	0.020*
Lymphocyte, ×10^9^/L^#^	1.0 (0.6–1.3)	1.2 (0.7–1.4)	0.17
Hemoglobin^&^	117.6 (17.7)	118.8 (19.3)	0.81
PLT^&^	203.4 (136.9)	219.6 (108.6)	0.75
ALT (U/L)^#^	41.0 (23.5–74.5)	53.0 (35.8–94.0)	0.19
AST (U/L)^#^	30.0 (22.0–64.0)	33.5 (23.0–61.0)	0.64
Albumin, g/L^&^	27.5 (4.8)	29.4 (5.6)	0.067
Total bilirubin^#^	17.0 (10.4–24.9)	13.0 (10.4–20.1)	0.15
Fasting blood glucose^&^	27.5 (4.8)	29.4 (5.6)	0.010*
BUN^&^	7.6 (5.0)	5.5 (2.7)	0.011*
Cr^&^	71.5 (34.3)	68.0 (21.8)	0.54
PT^&^	13.4 (1.4)	13.7 (1.7)	0.25
PCT^#^	2.1 (0.4–13.2)	1.4 (0.5–7.3)	0.72
CRP^&^	141.6 (91.1)	173.3 (100.6)	0.11
Clinical outcomes			
Length of hospital stay, days^&^	21.1 (10.0)	13.3 (7.1)	<0.001*
Admission to the ICU	1/50 (2.0)	1/50 (2.0)	1.00
Septic shock	2/50 (4.0)	2/50 (4.0)	1.00
In-hospital deaths	1/50 (2.0)	0/50 (0.0)	1.00

^#^: Median (IQR); ^&^: Mean (SD); Bold and *: *p* < 0.05; PLT: platelet count; ALT: alanine aminotransferase; AST: aspartate transaminase; BUN: blood urea nitrogen; Cr: creatinine; PCT: procalcitonin; CRP: C-reactive protein; ICU: intensive care unit.

### 3.2 Multivariate analysis of the clinical characteristics of patients with invasive and noninvasive KPLAs

**Figure 1:**
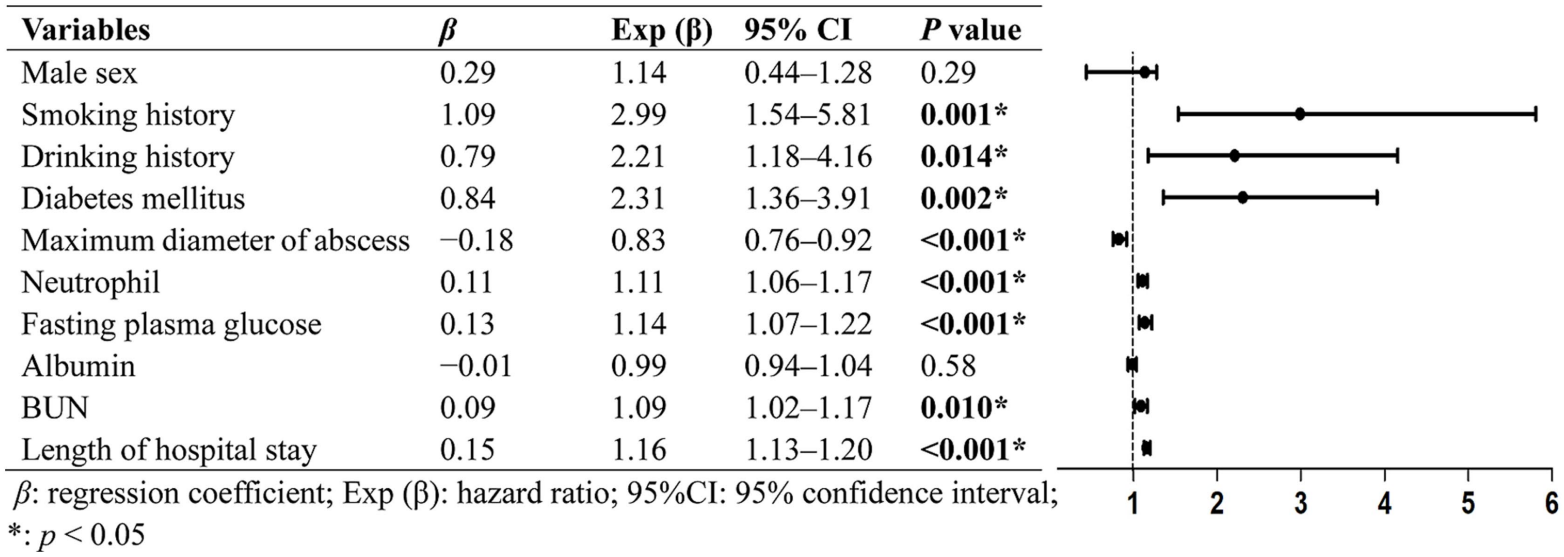
Multivariate analysis of the clinical characteristics associated with invasive KPLA. Variables with *p*-values <0.10 in univariate analysis were included in the multivariate logistics analysis. The results exhibited that diabetes mellitus, smoking history, drinking history, neutrophil count, fasting blood glucose, BUN, and length of hospital stay showed statistically significant positive associations with invasive KPLA. Whereas maximum diameter of abscess showed statistically significant negative associations with invasive KPLA.

Variables with *p*-values <0.10 were included in the multivariate logistics analysis (Figure 1), which showed that diabetes mellitus (odds ratio [OR], 2.31; 95% CI, 1.36–3.91; *p* = 0.002), smoking history (OR, 2.99; 95% CI, 1.54–5.81; *p* = 0.001), and drinking history (OR, 2.21; 95% CI, 1.18–4.16; *p* = 0.014) were identified as statistically independent risk factors for invasive KPLA. Additionally, neutrophil count, fasting blood glucose, BUN, and length of hospital stay showed positive associations with invasive KPLA, whereas maximum diameter of abscess showed negative associations with invasive KPLA. From a clinical perspective, neutrophil count, BUN, and length of hospital stay are more likely to be the consequences of invasive KPLA, whereas diabetes mellitus, high fasting blood glucose, and smoking and drinking history are more likely to be risk factors for invasive KPLA. The data on neutrophil counts, BUN values, maximum diameter of the abscesses, lengths of hospital stay, and laboratory tests related to glycemic control (fasting blood glucose, random blood glucose, and HbA1c) were shown in Supplementary Figure S1.

### 3.3 Phylogenetic analysis of *Klebsiella pneumoniae* strains

**Figure 2:**
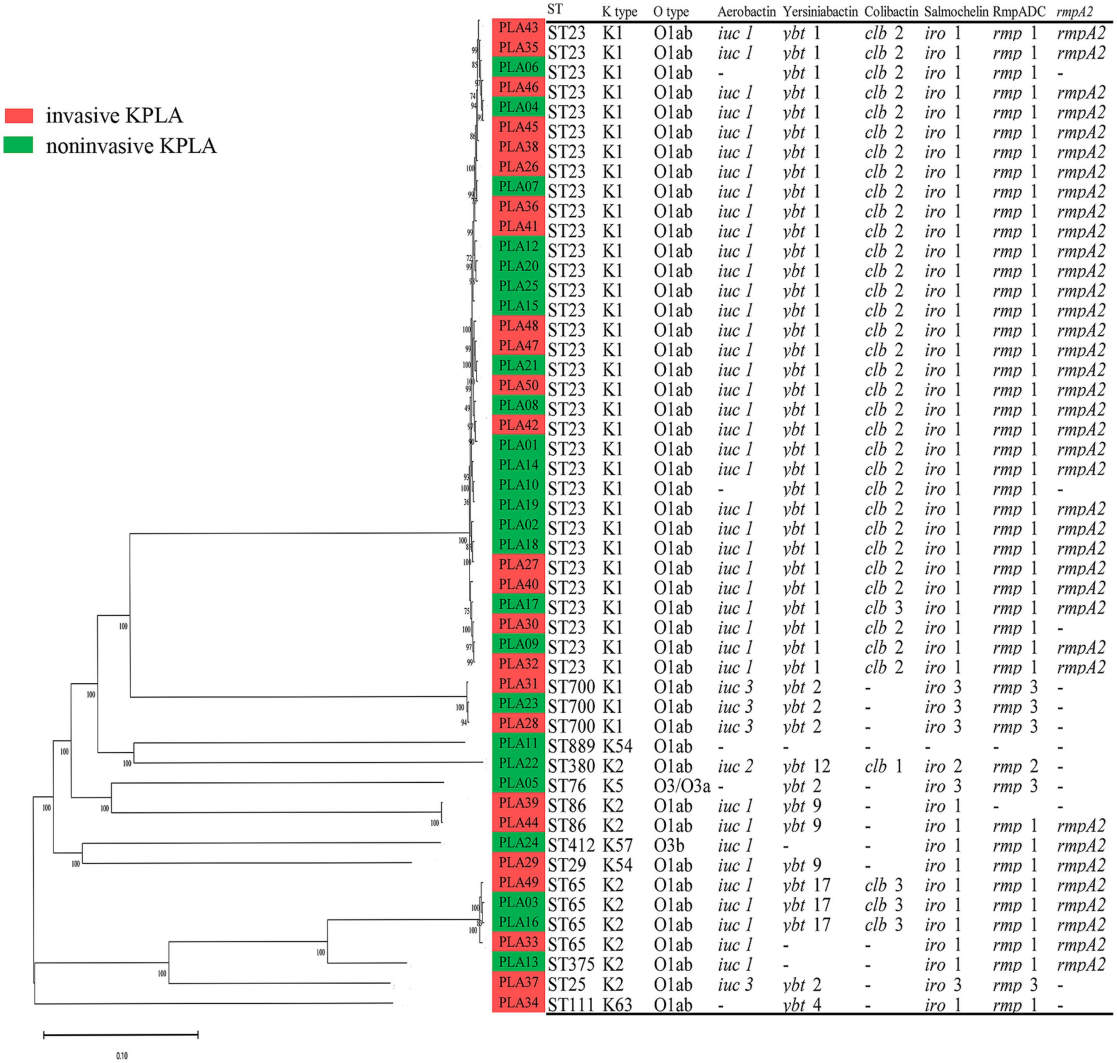
Phylogenetic analysis of 50 *K. pneumoniae* strains isolated from invasive KPLA (red) and noninvasive KPLA (green). The molecular characteristics (sequence type, K type, O type) and virulence determinants (Aerobactin, Yersiniabactin, Colibactin, Salmochelin, RmpADC, *rmpA2*) of the *K. pneumoniae* strains were also displayed. It showed that all strains except for one were hvKP, with ST23 and ST65 as the predominant sequence types. Besides, *K. pneumoniae* strains from invasive and noninvasive KPLAs were intermingled, without forming group-specific clusters.

The whole-genome sequences of 50 *K. pneumoniae* strains were used to construct a phylogenetic tree, as shown in Figure 2. It revealed that ST23 (33/50, 66.0%) was the predominant sequence type that was only found among K1 isolates. ST65 (8/50, 16.0%) was the second most prevalent sequence type that was only found among K2 isolates. K1 (36/50, 72.0%) and K2 (9/50, 18.0%) were the predominant capsular serotypes. Liver abscess was not caused by clonally related *K. pneumoniae* strains. Instead, these strains showed genetic differences as they contained different SNPs. All strains except for one (PLA11) carried multiple virulence genes and was classified as hvKP. Additionally, *K. pneumoniae* strains from patients with invasive and noninvasive KPLAs were intermingled as showed by the phylogenetic tree, without forming group-specific clusters.

### 3.4 Microbiological and molecular characteristics of *Klebsiella pneumoniae* strains from patients with invasive and noninvasive KPLAs

The multilocus sequence types, capsular serotypes, virulence determinants, string test results, and antimicrobial resistance patterns of *K. pneumoniae* strains isolated from patients with invasive and noninvasive KPLAs were shown in Table 2. There was no significant difference in these characteristics between the two groups. Approximately 94% of the *K. pneumoniae* strains showed a hypermucoviscous phenotype. In addition, 49 *K. pneumoniae* strains were susceptible to the antimicrobials tested, including piperacillin, cefazolin, cefepime, ceftriaxone, ceftazidime, ciprofloxacin, levofloxacin, imipenem, meropenem, gentamicin, amikacin, and aztreonam. PLA27 was resistant to cephalosporins and quinolones, which was consistent with the presence of the *CTX-M-3*, *aac(3)-IId*, and *qnrS1* resistance genes in its genome.

**Table 2:** Microbiological and molecular characteristics of *K. pneumoniae* strains isolated from patients with invasive and noninvasive KPLAs.

Characteristic	Invasive KPLA (*n* = 25)	Noninvasive KPLA (*n* = 25)	*P* value
Multilocus sequence typing			
ST23	16 (64.0)	17 (68.0)	0.77
ST65	2 (8.0)	2 (8.0)	1.00
ST700	2 (8.0)	1 (4.0)	1.00
Other	5 (20.0)	5 (20.0)	1.00
Capsular serotypes			
K1	18 (72.0)	18 (72.0)	1.00
K2	5 (20.0)	4 (16.0)	1.00
K54	1 (4.0)	1 (4.0)	1.00
Other	1 (4.0)	2 (8.0)	1.00
String test	23 (92.0)	24 (96.0)	1.00
Virulence score	4.5 (0.9)	4.2 (1.5)	0.36
Virulence determinants			
Yersiniabactin	24 (96.0)	22 (88.0)	0.61
Colibactin	17 (68.0)	20 (80.0)	0.33
Aerobactin	24 (96.0)	21 (84.0)	0.35
Salmochelin	25 (100.0)	24 (96.0)	1.00
RmpADC	24 (96.0)	24 (96.0)	1.00
RmpA2	19 (76.0)	19 (76.0)	1.00
Resistance score	0.0 (0.0)	0.0 (0.2)	0.32
Antimicrobial resistance			
Piperacillin	1 (4.0)	0 (0.0)	1.00
Cefazolin	1 (4.0)	0 (0.0)	1.00
Cefepime	1 (4.0)	0 (0.0)	1.00
Ceftriaxone	1 (4.0)	0 (0.0)	1.00
Ceftazidime	0 (0.0)	0 (0.0)	1.00
Ciprofloxacin	1 (4.0)	0 (0.0)	1.00
Levofloxacin	1 (4.0)	0 (0.0)	1.00
Imipenem	0 (0.0)	0 (0.0)	1.00
Meropenem	0 (0.0)	0 (0.0)	1.00
Gentamicin	1 (4.0)	0 (0.0)	1.00
Amikacin	0 (0.0)	0 (0.0)	1.00
Aztreonam	1 (4.0)	0 (0.0)	1.00

### 3.5 Virulence genes and SNPs of *Klebsiella pneumoniae* strains isolated from patients with invasive and noninvasive KPLAs

Analysis of the virulence genes of *K. pneumoniae* strains isolated from patients with invasive and noninvasive KPLAs (Table 3) revealed no significant differences in the frequencies of genes related to adherence (type 3 fimbriae, type I fimbriae, and type IV pili), iron uptake (aerobactin, siderophore, salmochelin, yersiniabactin, and iron transport), nutritional factors (allantoin utilization), regulation (RcsAB and RmpA), secretion system (T6SS I, T6SS II, and T6SS III), and toxin (colibactin) between the two groups. In addition, analysis of the SNPs of *K. pneumoniae* strains isolated from patients with invasive and noninvasive KPLAs (Supplementary Table S2) revealed no group-specific SNPs. Besides, analysis of SNP frequencies showed no significant differences between the two groups.

**Table 3:** Virulence genes of *K. pneumoniae* strains isolated from patients with invasive and noninvasive KPLAs.

Virulence genes	Invasive KPLA (*n* = 25)	Noninvasive KPLA (*n* = 25)	*P* value	Virulence genes	Invasive KPLA (*n* = 25)	Noninvasive KPLA (*n* = 25)	*P* value
Type 3 fimbriae				Allantoin utilization			
*mrkA*	25	25	1.00	*allA*	16	18	0.54
*mrkB*	25	25	1.00	*allB*	16	18	0.54
*mrkC*	25	25	1.00	*allC*	16	18	0.54
*mrkD*	25	25	1.00	*allD*	16	18	0.54
*mrkF*	25	25	1.00	*allR*	16	18	0.54
*mrkH*	25	25	1.00	*allS*	16	18	0.54
*mrkI*	25	25	1.00	Regulation			
*mrkJ*	25	25	1.00	*rcsA*	25	25	1.00
Type I fimbriae				*rcsB*	25	25	1.00
*fimA*	23	24	1.00	*RmpA*	0	3	0.24
*fimB*	25	25	1.00	T6SS I			
*fimC*	25	24	1.00	*clpV/tssH*	22	22	1.00
*fimD*	25	25	1.00	*dotU/tssL*	23	23	1.00
*fimE*	25	24	1.00	*hcp/tssD*	22	22	1.00
*fimF*	25	24	1.00	*icmF/tssM*	23	23	1.00
*fimG*	25	24	1.00	*impA/tssA*	17	20	0.33
*fimH*	25	24	1.00	*ompA*	22	22	1.00
*fimI*	25	24	1.00	*sciN/tssJ*	23	23	1.00
*fimK*	25	24	1.00	*tle1*	1	0	1.00
AcrAB				*tli1*	6	3	0.46
*acrA*	25	25	1.00	*tssF*	25	25	1.00
*acrB*	25	25	1.00	*tssG*	23	23	1.00
Type IV pili				*vasE/tssK*	22	23	1.00
*pliW*	3	2	1.00	*vgrG/tssI*	22	22	1.00
Aerobactin				*vipA/tssB*	23	22	1.00
*iucA*	24	21	0.35	*vipB/tssC*	22	22	1.00
*iucB*	24	21	0.35	T6SS II			
*iucC*	24	21	0.35	*clpV*	25	25	1.00
*iucD*	24	21	0.35	*dotU*	0	1	1.00
*iutA*	25	25	1.00	*icmF*	0	1	1.00
Ent siderophore				*impF*	0	1	1.00
*entA*	25	25	1.00	*impH*	0	1	1.00
*entB*	25	25	1.00	*impJ*	0	1	1.00
*entC*	25	25	1.00	*ompA*	0	1	1.00
*entD*	25	25	1.00	*sciN*	0	1	1.00
*entE*	25	25	1.00	*vasA/impG*	0	1	1.00
*entF*	25	25	1.00	*vgrG*	0	1	1.00
*entS*	25	25	1.00	T6SS III			
*fepA*	25	25	1.00	*dotU*	25	25	1.00
*fepB*	25	25	1.00	*icmF*	24	23	1.00
*fepC*	25	25	1.00	*impA*	25	25	1.00
*fepD*	25	25	1.00	*impF*	25	25	1.00
*fepG*	25	25	1.00	*impG*	23	23	1.00
*fes*	25	25	1.00	*impH*	25	25	1.00
Salmochelin				*impJ*	25	25	1.00
*iroB*	25	24	1.00	*lysM*	0	0	1.00
*iroC*	25	24	1.00	*ompA*	25	25	1.00
*iroD*	25	24	1.00	*sciN*	25	25	1.00
*iroE*	25	25	1.00	*vgrG*	23	24	1.00
*iroN*	25	25	1.00	Colibactin			
Yersiniabactin				*clbA*	0	3	0.24
*fyuA*	24	22	0.61	*clbB*	17	20	0.33
*irp1*	23	22	1.00	*clbC*	17	20	0.33
*irp2*	24	22	0.61	*clbD*	0	3	0.24
*ybtA*	24	22	0.61	*clbE*	13	13	1.00
*ybtE*	24	22	0.61	*clbF*	17	20	0.33
*ybtP*	24	22	0.61	*clbG*	17	20	0.33
*ybtQ*	24	22	0.61	*clbH*	17	20	0.33
*ybtS*	24	21	0.35	*clbI*	16	20	0.21
*ybtT*	24	22	0.61	*clbJ*	17	20	0.33
*ybtU*	24	22	0.61	*clbK*	17	19	0.53
*ybtX*	24	22	0.61	*clbL*	17	20	0.33
Iron transport				*clbM*	17	20	0.33
*sitA*	6	5	0.73	*clbN*	17	20	0.33
*sitC*	15	16	0.77	*clbO*	17	20	0.33
*sitD*	14	16	0.56	*clbP*	17	20	0.33
				*clbQ*	16	19	0.36
				*clbS*	3	0	0.24

## 4 Discussion

KPLA is caused by hvKP that initially colonizes the gastrointestinal tract of the host. From there, it translocates across the intestinal barrier via the hepatic portal vein into the liver, where it causes liver abscesses. Liver Kupffer cells and macrophages have been shown to kill hvKp *in vitro* (Hoh et al., 2019). However, the capsule of *K. pneumoniae* protects it from being captured by liver Kupffer cells, as evidenced by the ability of certain high-virulence capsular serotypes to completely block Kupffer cell capture, whereas the low-virulence counterparts confer only partial protection against Kupffer cell capture (Huang et al., 2022). Risk factor analysis for PLA indicates that men are at a higher risk of developing PLA than women. Besides, patients with diabetes, especially those with poor glycemic control and high BMI had a significantly higher risk of developing PLA (Wang et al., 2023). Certain comorbidities, such as liver transplantation and malignancy, increase the risk of developing PLA (Kaplan et al., 2004).

Serious cases of KPLA may be accompanied with metastatic infections, such as endophthalmitis and lung abscess. Despite its low prevalence, invasive KPLA is a severe damaging and difficult-to-treat disease. Such invasive infections may result in significant disability and high mortality in patients with KPLA. Therefore, the present study aimed to determine the risk factors associated with invasive KPLA. Our results showed that diabetes mellitus, drinking history, and small diameter of abscess were significant risk factors for invasive KPLA. This is consistent with previous studies which revealed that diabetes mellitus, alcoholism, and maximal diameter of abscess < 55 mm were independent risk factors for metastatic infection among patients with KPLA (Chen et al., 2006; Yoon et al., 2014; Chang et al., 2015). These three factors have been reported in more than one study, suggesting that they are important risk factors for invasive KPLA. However, other risk factors, such as smoking history, high fasting blood glucose level, high neutrophil count, high BUN level, long length of hospital stay, were first reported as independent risk factors invasive KPLA, which need to be confirmed by clinical studies in the future.

Diabetes mellitus and high fasting blood glucose levels, which indicate poor glycemic control, were risk factors for invasive KPLA. Moreover, patients with invasive KPLA showed higher random blood glucose and HbA1c levels than those with noninvasive KLPA. These results were consistent with those of previous studies showing that diabetes is a significant risk factor for the development of endogenous endophthalmitis (Lin et al., 2006; Sheu et al., 2011). Besides, another study showed that fasting blood glucose is related with invasion syndrome in patients with diabetes complicated with KPLA (Feng et al., 2024). On one hand, high glucose may up-regulate the levels of *rmpA* and *ompA* in hvKP and enhance its resistance to serum killing, thus increasing its virulence (Tang et al., 2023). On the other hand, high glucose levels may impair neutrophil phagocytosis of K1 and K2 *K. pneumoniae* (Lin et al., 2006), providing a reasonable explanation for the high incidences of invasive KPLA in patients with poor glycemic control.

To the best of our knowledge, this is the first study to report that cigarette smoking is an independent risk factor for invasive KPLA. Similar associations have been reported in certain respiratory infections. For example, cigarette smoking prolongs the inflammation caused by influenza A infection and delays the clearance of the infection in mice (Vlasma et al., 2024). Cigarette smoking also attenuates mice’s nasal response to *S. pneumoniae* and predisposes them to invasive pneumococcal disease (Shen et al., 2016). Another study reported that smoking increases the virulence of *P. aeruginosa* and induces resistance to neutrophil killing, resulting in a greater capacity to cause invasive disease (Chien et al., 2020). Cigarette smoking can modify neutrophil chemotaxis, and induce neutrophil extracellular trap (NET) formation (White et al., 2018; Wang et al., 2025); whereas, how cigarette smoking causes aggressive KPLA remains elusive and needs further research.

In addition, alcohol drinking was found to be a significant predictor of invasive KPLA. Previous research found that alcohol consumption was associated with abnormal neutrophil traps and reduced lipopolysaccharide-induced NET formation, which contribute to liver injury and sepsis severity (Bukong et al., 2018). Additionally, alcohol can induce decreased neutrophil phagocytosis and oxidative burst to *E. coli* (Khan et al., 2023). Therefore, we speculate that alcohol drinking impairs host defense functions by decreasing the phagocytic activity of neutrophils, thereby making patients more susceptible to invasive KPLA. Further experimental studies are needed to validate the hypothesis.

Previous studies have demonstrated that the hypervirulent capsular serotypes K1 and K2 of *K. pneumoniae* are the major serotypes responsible for KPLA (Chuang et al., 2016). These serotypes are hypermucoviscous and resistant to serum bactericidal activity and neutrophil phagocytosis (Al-Busaidi et al., 2024). These results are consistent with those of our study, which identified hvKP as the major pathogen responsible for both invasive and noninvasive KPLAs, with K1 serotype ST23 being the most prominent. However, we found no significant differences in the microbiological, molecular, and virulence gene characteristics as well as the SNPs of *K. pneumoniae* strains isolated from patients with invasive and noninvasive KPLAs. These results indicate that both invasive and noninvasive KPLAs are caused by hvKP, and bacterial virulence characteristics are not a critical factor for the development of invasive KPLA.

This present study has significant impact on the clinical management of KPLA. Firstly, results of the study may aid clinicians in early identification of KPLA patients with extrahepatic metastasis infection, so as to initiate optimized therapy as soon as possible to reduce the rate of mortality and adverse events. Secondly, poor glycemic control, smoking history, and drinking history were demonstrated to be significantly associated with increased risk of developing invasive KPLA, which indicates that effective glycemic control, smoking and alcohol quit should be recommended for patients with high risk of developing KPLA. However, this study has several limitations. Small sample size due to the relative low prevalence of invasive KPLA, may result in bias of the study. Additionally, accuracy of the findings may be affected due to missing data problems. Moreover, in-depth investigation into the molecular mechanisms underlying the findings in this study are warranted in the future.

## 5 Conclusion

Physicians should be vigilant for the possibility of invasive KPLA when patients with PLA are accompanied with conditions such as diabetes mellitus, smoking history, drinking history, high fasting blood glucose level, high neutrophil count, high BUN level, long length of hospital stay, and small diameter of abscess. In the present study, the bacterial molecular and virulence characteristics did not differ significantly between the two groups. Overall, our results indicate that patients’ contaminant conditions, such as poor glycemic control, smoking history, and drinking history, rather than bacterial virulence, contribute to the development of invasive KPLA. This study is of great significance for the early identification and clinical management of invasive KPLA.

## Data Availability

The raw sequence data reported in this paper have been deposited in China National Center for Bioinformation/Beijing Institute of Genomics, Chinese Academy of Sciences (GSA: CRA026012) that are publicly accessible at https://ngdc.cncb.ac.cn/gsa.

## Footnotes

^1^ https://github.com/ablab/spades?tab=License-1-ov-file

^2^ https://pathogen.watch/

^3^ http://www.mgc.ac.cn/VFs/
