# Supplementary material for: Poor glycemic control and smoking and drinking history rather than bacterial virulence contribute to the development of invasive Klebsiella pneumoniae liver abscess: a case–control study in Northeast China
Source: Front Microbiol. 2025 Aug 26;16:1650703. doi: 10.3389/fmicb.2025.1650703 (PMC12417397; doi:10.3389/fmicb.2025.1650703)
Supplement: Supplementary file 1 [file Data_Sheet_1.docx]

**Supplementary files for the manuscript:** Poor glycemic control and smoking and drinking history rather than bacterial virulence contribute to the development of invasive *Klebsiella pneumoniae* liver abscess: a case-control study in northeast China.

**Legend of Tables:**

**Supplementary Table S1**: Clinical and microbiological characteristics of 100 PLA patients.

**Supplementary Table S2**: SNP characteristics of 50 PLA patients.

**Legend of Figures:**

**Supplementary Figure S1**: Comparison of neutrophil counts (A), BUN values (B), maximum diameter of the abscesses (C), lengths of hospital stay (D), fasting blood glucose (E), random blood glucose (F), and HbA1c (G) between invasive and noninvasive KPLA, which showed statistically difference.
