# Supplementary figures and images for: Poor glycemic control and smoking and drinking history rather than bacterial virulence contribute to the development of invasive Klebsiella pneumoniae liver abscess: a case–control study in Northeast China
Source: Front Microbiol. 2025 Aug 26;16:1650703. doi: 10.3389/fmicb.2025.1650703 (PMC12417397; doi:10.3389/fmicb.2025.1650703)

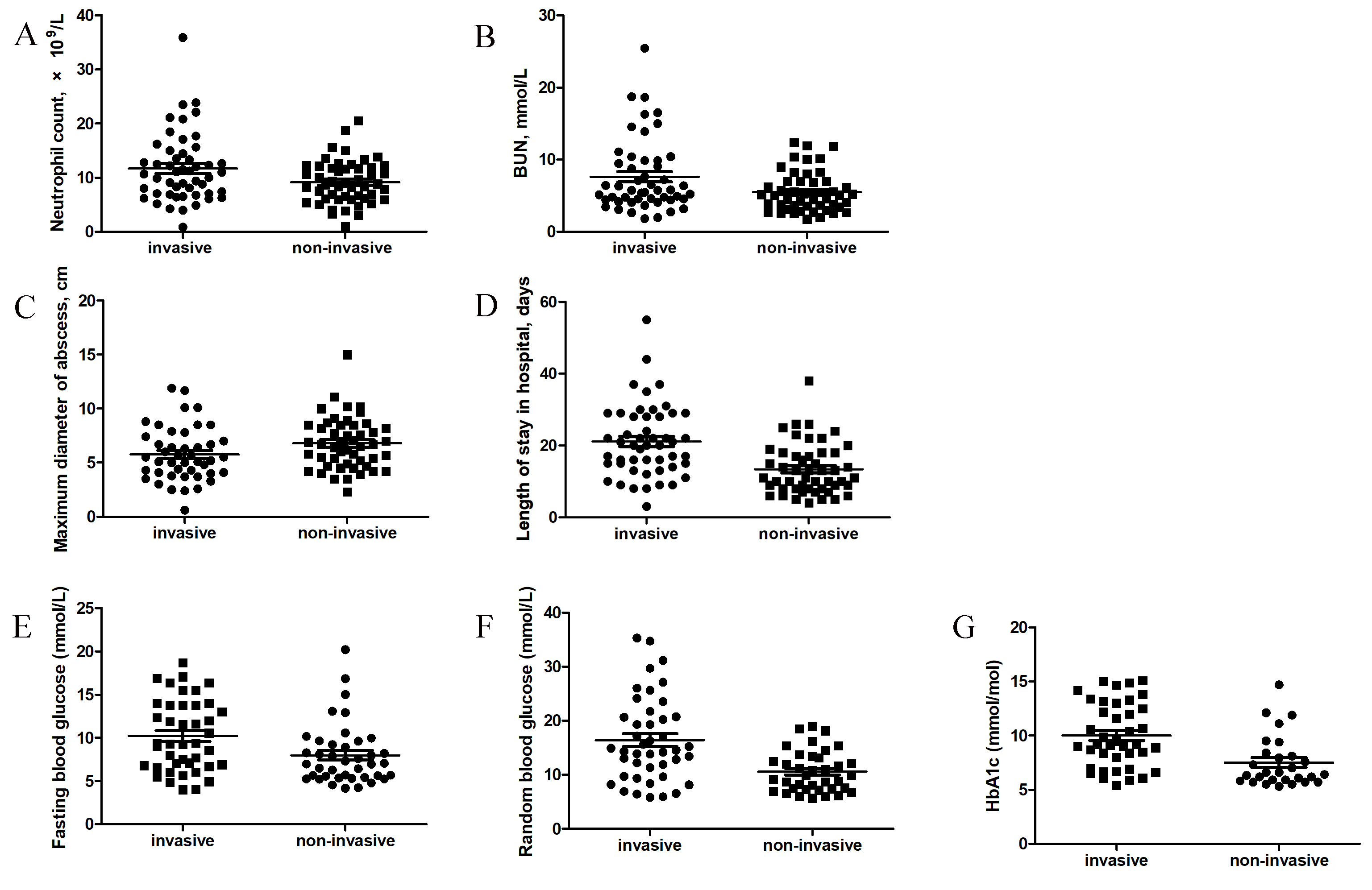

Supplement: Supplementary file 2 [file Image_1.tif]
